# Nicotine exposure impairs germ cell development in human fetal ovaries cultured *in vitro*

**DOI:** 10.18632/aging.101492

**Published:** 2018-07-12

**Authors:** Shun-Feng Cheng, Xun-Si Qin, Ze-Li Han, Xiao-Feng Sun, Yan-Ni Feng, Fan Yang, Wei Ge, Lan Li, Yong Zhao, Massimo De Felici, Shu-Hua Zou, Yi Zhou, Wei Shen

**Affiliations:** 1 College of Life Sciences, Institute of Reproductive Sciences, Qingdao Agricultural University, Qingdao 266109, China; 2 The First Affiliated Hospital of Chinese PLA General Hospital, Beijing 100039, China; 3 Department of Biomedicine and Prevention, University of Rome ‘Tor Vergata’, 00133 Rome, Italy; 4 Center for Reproductive Medicine, Qingdao Women’s and Children’s Hospital, Qingdao University, Qingdao 266034, China; * Equal contribution

**Keywords:** nicotine, human fetal ovary, germ cells, apoptosis, DNA damage

## Abstract

In the present paper, we found that human fetal ovaries (at ~16 weeks) express the transcripts for several subunits of the nicotinic acetylcholine receptor (nAChR). Exposure to the drug *in vitro* resulted in the marked increase of apoptosis in the ovaries in a time and dose-dependent manner. Evidence that adverse nicotine effects are potentially due to an increased level of reactive oxygen species (ROS) and consequent DNA damage, both in the ovarian somatic cells and germ cells, are reported. After 4 days of culture, exposure to 1 mM and 10 mM nicotine caused a 50% and 75% decrease, respectively, in the number of oogonia/oocytes present in the fetal ovaries. These results represent the first indication that nicotine may directly cause apoptosis in cells of the fetal human ovary and may lead to a reduction of the ovarian reserve oocytes and consequent precocious menopause in mothers smoking during pregnancy.

## Introduction

The adverse effects of maternal tobacco smoke on fetuses have been well documented for more than three decades and remains one of the main preventable causes of perinatal complications. Complications include low birth weight, preterm delivery, stillbirth, high perinatal mortality and birth defects of the offspring [1]. Cigarette smoke is composed of more than 4,000 chemical compounds including hundreds of potential reproductive toxicants and carcinogens that have been associated with a variety of adverse reproductive outcomes [2]. Evidence is accumulating that components present in cigarette smoke can affect the prenatal development of the reproductive organs including the ovaries in exposed offspring [3-7].

During the prenatal period critical processes involving female gametogenesis occur. In humans, around the end of the 3^rd^ week of development, a small number (between 50-100) of gamete precursors known as primordial germ cells (PGCs) are set aside in an extra embryonic location, the yolk sac wall, and begin to migrate towards the gonadal ridges (GRs) [8]. After entering into the GRs (7-9 weeks gestation), PGCs, now called oogonia, undergo several rounds of mitotic division leading to a large increase in their numbers [9]. Mitotic divisions of oogonia ends around the 18^th^ week of gestation, reaching a peak number of germ cells at 6-7 million. From about 9^th^ week, the first oogonia begin to enter meiosis [10]. By entering meiosis, female germ cells lose the ability to divide mitotically and are now termed oocytes. At 18-20 weeks, some oocytes, upon reaching the diplotene stage, are surrounded by flattened pregranulosa cells to form primordial follicles, some of which soon develop into primary follicles. At birth the majority of oocytes are arrested at the diplotene stage and enclosed in a primordial follicle [11,12]. During late gestation a large number of oogonia and oocytes undergo cell death which reduces the germ cell population to ~1-2 million at birth. Therefore, the finite ovarian follicle pool, termed the ovarian reserve, is established before birth, resulting from the balance of oogonia proliferation and oogonia and oocyte death. All gametogenesis processes reported above are potentially vulnerable to environmental pollutants that can impact the fertility of females postnatally [13,14].

Concerning the exposure to cigarette smoke, a reduction of about 20% of the ovarian reserve [15] and precocious menopause in smoking women have been reported [4,16,17]. In addition, in some patients a decrease in the number of oocytes and somatic cells in the ovary [3,6,9], reduced fertility, and earlier menopause [18] have been associated with mothers smoking during pregnancy, although contradictory results have also been reported [5,7].

Several studies performed in animal models identified polycyclic aromatic hydrocarbons (PAHs) as the components of cigarette smoke causing adverse effects on female reproduction. In fact, it has been shown that in rodents that benzo[α]pyrene (BαP), a main component of cigarette smoke PAHs, impairs follicular growth *in vitro* [19], while *in vivo* exposure of adult females leads to a loss of primordial follicles [16,20-22], through the induction of apoptosis [16]. Moreover, in utero exposure to BαP has been reported to result in a reduced pool of primordial follicles or infertility in mice [23-26]. Further evidence is provided through studies showing *in vitro* exposure of mouse embryonic ovaries to 9,10-dimethylbenzen(a)anthracene-3,4-dihydrodiol (DMMA-DHD), a PAH known to bind and activate the aromatic hydrocarbon receptor (AHR) present in ovarian somatic and germ cells of several species including human [27-30], induced apoptotic death of fetal germ cells through direct activation of *Bax* expression [31]. Interestingly, in chickens, the interaction PAH-AHR suppresses PGC meiosis independently from *Bax* [32]. Finally, reduced germ cell proliferation was observed after exposure of first trimester human fetal ovaries to PAHs [8].

Nicotine, the main active alkaloid component of cigarette tobacco, is considered a major teratogenic chemical able to perturb embryonic development [1,33,34]. Nicotine can quickly cross the placenta to reach the embryo and accumulates in fetal blood and amniotic fluid [35]. Nicotine or its main metabolite cotinine exert their effects by activating nicotinic acetylcholine receptors (nAChRs), which are transmembrane ligand-gated ion channels consisting of five subunits. When activated nAChRs have been shown to increase ion influx, mainly involving Ca^2+^ [36]. Elevation of intracellular Ca^2+^ concentration often has impacts on a variety of intracellular signaling and organelle functions [37]. For instance, though elevation of intracellular Ca^2+^, nicotine may increase the formation of reactive oxygen species (ROS) leading to oxidative stress in the cell. ROS has been demonstrated to be one of the major factors causing DNA damage and leading to apoptosis [34,38-40]. Conversely, through activation of the protein kinase B (PKB/AKT) and extracellular regulated protein kinases (ERK) signaling pathways, nicotine also able to prevent apoptosis in certain cell types. The variety of effects of nicotine on adult physiology and tumorigenesis have been extensively studied [33,41]. Some data from animal studies provide evidence that nicotine exposure potentially has adverse effects on female reproduction [42,43]. For example, *in utero* exposure to nicotine causes impaired fertility, altered ovarian steroid hormone and protein levels, and an increased numbers of atretic follicles in adult female rat offspring [19,44]. Of particular note, exposure to the drug during fetal, neonatal and adult age induces altered morphology and apoptosis in mouse and rat ovaries [44-46]. Petrik and coll. (2009) [45], found nAChR-2 and nAChR-7 expression in ovarian tissues and isolated granulosa cells and suggested that one mechanism by which nicotine may cause the folliculogenesis defects observed in adult female rats was through the induction of apoptosis in granulosa cells and/or oocytes via activation of the receptors.

The present study was designed to investigate whether exposure to nicotine induces direct toxicity during the development of human ovaries. To this aim, ovaries obtained from fetuses during the second trimester of pregnancy were cultured *in vitro* for four days in the presence of various nicotine concentrations and analyzed for several morphological and molecular parameters in order to detect developmental defects.

## RESULTS

### Nicotine exposure induced cell apoptosis

**Figure 1:**
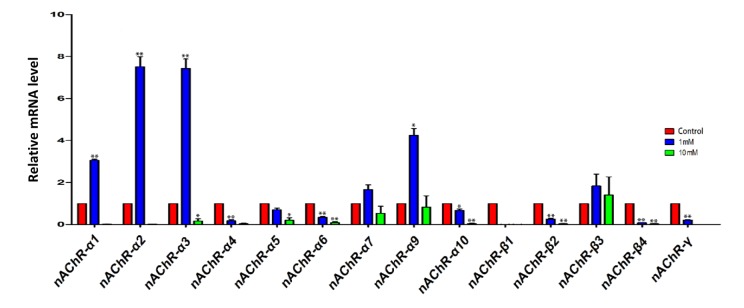
**qRT-PCR analysis of nicotinic acetylcholine receptor (nAChRs) subunit mRNA in fetal ovaries cultured for 4 days in the presence of 1mM or 10mM nicotine.** The expression levels were normalized to that of *Gapdh* gene as a control. All experiments were repeated at least three times. Results are presented as mean ± SD in comparison to control. (*) and (**) indicate significant (P < 0.05) and highly significant (P < 0.01) difference, respectively.

In a first series of experiments, we investigated the expression of nicotinic acetylcholine receptors (nAChRs) in the ovary using qRT-PCR. The results showed that the ovaries expressed the transcripts of various subunits of nAChRs, and that exposure for 4 days to 1 mM nicotine significantly increased the mRNA level of some subunits, namely nAChRα1, 2, 3 and 9, in comparison to control, while 10mM nicotine caused, almost invariably, a decrease of the transcript levels of most of the subunits (Figure 1).

**Figure 2:**
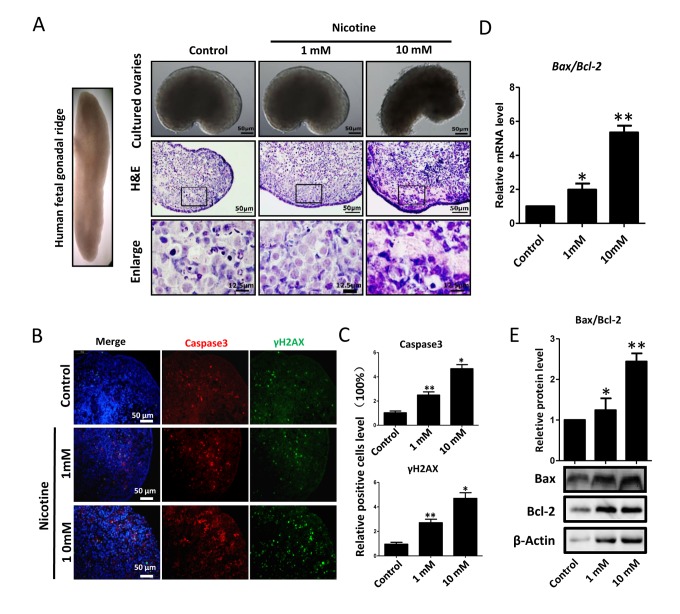
**Dose-dependent nicotine induction of apoptosis in fetal ovaries cultured for 4 days.** (**A**) Ovaries cultured without (control) and with 1 mM or 10 mM nicotine; note altered ovary morphology at 10mM nicotine and representative H&E histological sections of the ovaries; (**B**) IF for Caspase3 and ɤH2AX in tissue sections of ovaries cultured without (control) and with 1mM or 10mM nicotine; (**C**) Relative percentage of Caspase3 and ɤH2AX positive cells in ovaries cultured without (control) and with 1mM or 10mM nicotine; (**D**) *Bax/Bcl2* mRNA ratio in samples extracts from ovaries cultured without (control) and with 1mM or 10mM nicotine. The expression level was normalized to that of *Gapdh*. (**E**) Increased BAX/BCL2 protein ratio in nicotine exposed ovaries in comparison with control. All experiments were repeated at least three times. Changes are presented as mean ± SD. (*) and (**) indicate significant (P < 0.05) and highly significant (P < 0.01) difference, respectively.

**Figure 3:**
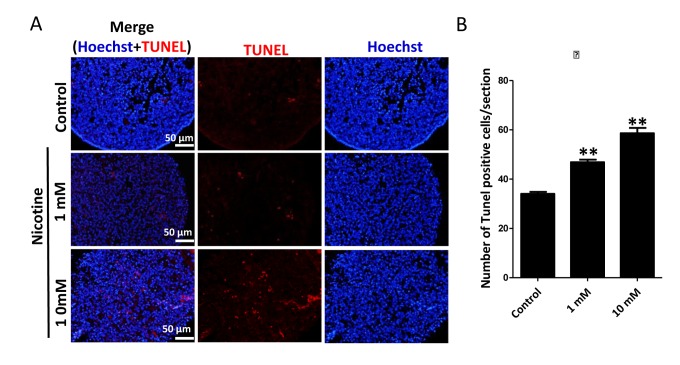
**Nicotine exposure increases apoptosis in cultured fetal ovaries.** (**A**) TUNEL-stained ovarian tissues cultured *in vitro* for 4 days. (**B**) Percentages of TUNEL positive cells in ovary tissue sections. All experiments were repeated at least three times. Results are presented as mean ± SD. (*) and (**) indicate significant (P < 0.05) and highly significant (P < 0.01) difference, respectively.

Next, we found that whereas ovaries cultured in the presence of 1mM nicotine for 4 days did not displayed evident morphological alterations, those exposed to the 10 mM drug showed loss of tissue integrity and smaller size (Figure 2A). Similarly, histological sections did not reveal morphological changes in the ovaries incubated in 1mM nicotine whereas in ovaries exposed to 10mM nicotine, most of the cells showed morphological alterations including an irregular shape and contracted and condensed cytoplasm, compatible with advanced stages of apoptosis and/or secondary necrosis (Figure 2A). However, using immunofluorescence (IF) we found a significant 2-3 and 4-5 fold increase of the numbers of cells positive for activated Caspase 3, indicating ongoing apoptosis, in ovaries after 4 days of culture in the presence of 1mM nicotine and 10mM, respectively (Figure 2B, C and Figure S1, S2). As a note, only a few cells positive for activated Caspase 3 were detected in the control ovaries (Figure 2B, C). The occurrence of apoptosis in the nicotine treated ovaries was supported by the increase of *Bax/Bcl2* mRNA and protein ratio evaluated by qRT-PCR (Figure 2D) and WB (Figure 2E). In addition, we investigated the extent of apoptosis in ovaries cultured in the presence or absence of nicotine using TUNEL staining. The results showed that the number of TUNEL positive cells in 1 mM and 10 mM nicotine treated ovaries for 4 days were significantly increased in comparison with controls (Figure 3).

### Degeneration of proteins and genes induced by nicotine in ovary cultured *in vitro*

**Figure 4:**
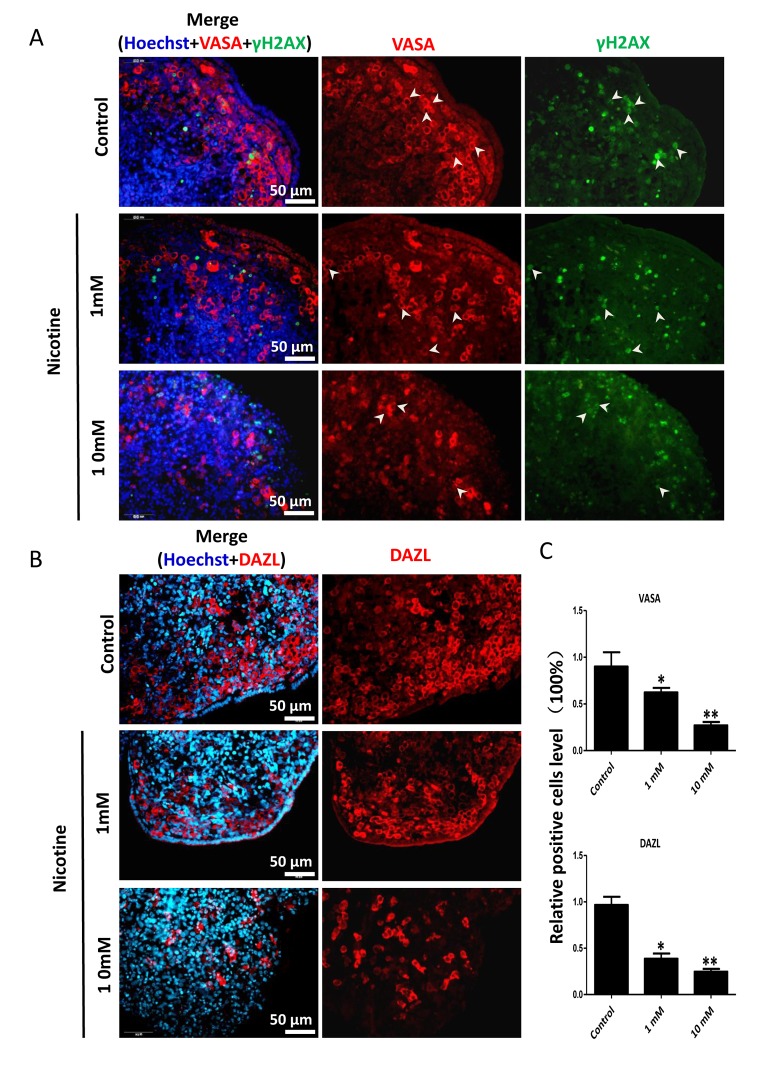
**Dose-dependent decrease or increase of the number of germ cell (VASA, DAZL) and of γH2AX positive cells, respectively, in nicotine treated fetal ovaries cultured for 4 days.** (**A**) Representative IF images of ovarian tissue sections for VASA and γH2AX; note that only a subset of the γH2AX positive cells were also VASA positive (arrow heads); (**B**) Representative IF images of ovarian tissue sections for DAZL; (**C**) Relative percentage of VASA and DAZL positive cells of ovaries cultured without (control) and with 1mM or 10mM nicotine. All experiments were repeated at least three times. (*) and (**) indicate significant (P < 0.05) and highly significant (P < 0.01) difference, respectively.

**Figure 5:**
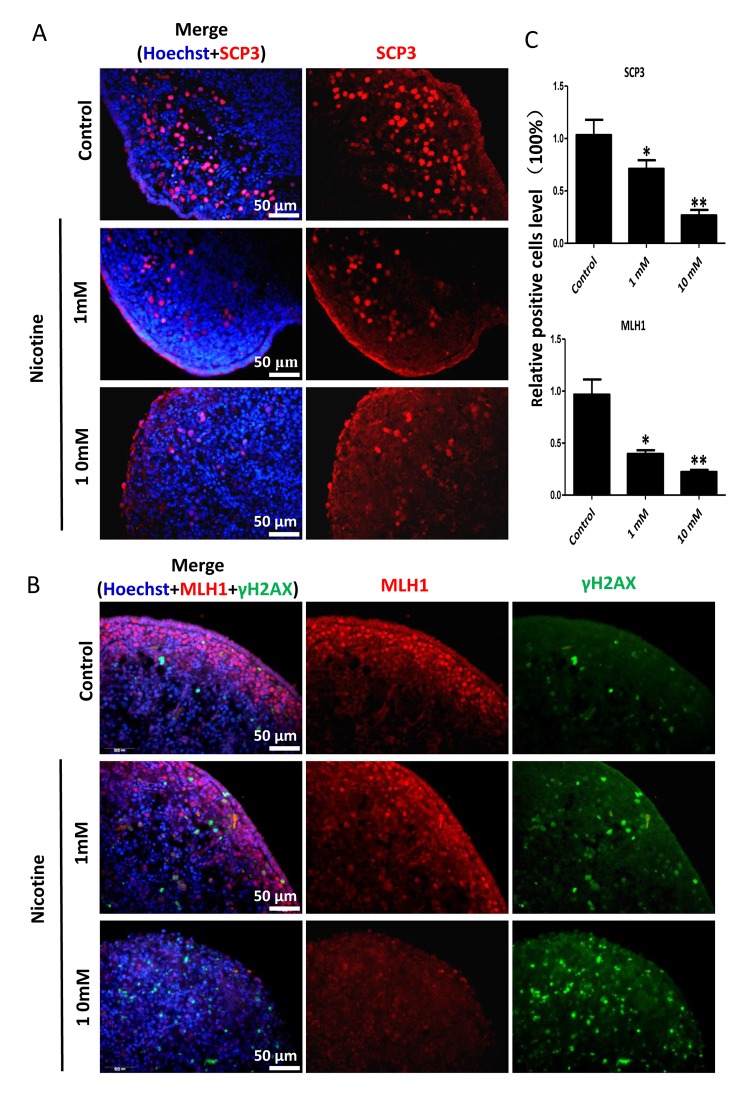
**Dose-dependent decrease or increase of the number of meiotic germ cells (SCP3, MLH1) and of γH2AX positive cell, respectively, in nicotine treated fetal ovaries cultured for 4 days.** (**A**) Representative IF images of ovarian tissue sections for SCP3; (**B**) representative IF images of ovarian tissue sections for MLH1 and γH2AX; (**C**) Relative percentage of SCP3 and MLH1 positive cells of ovaries cultured without (control) and with 1mM or 10mM nicotine. All experiments were repeated at least three times. (*) and (**) indicate significant (P < 0.05) and highly significant (P < 0.01) difference, respectively.

**Figure 6:**
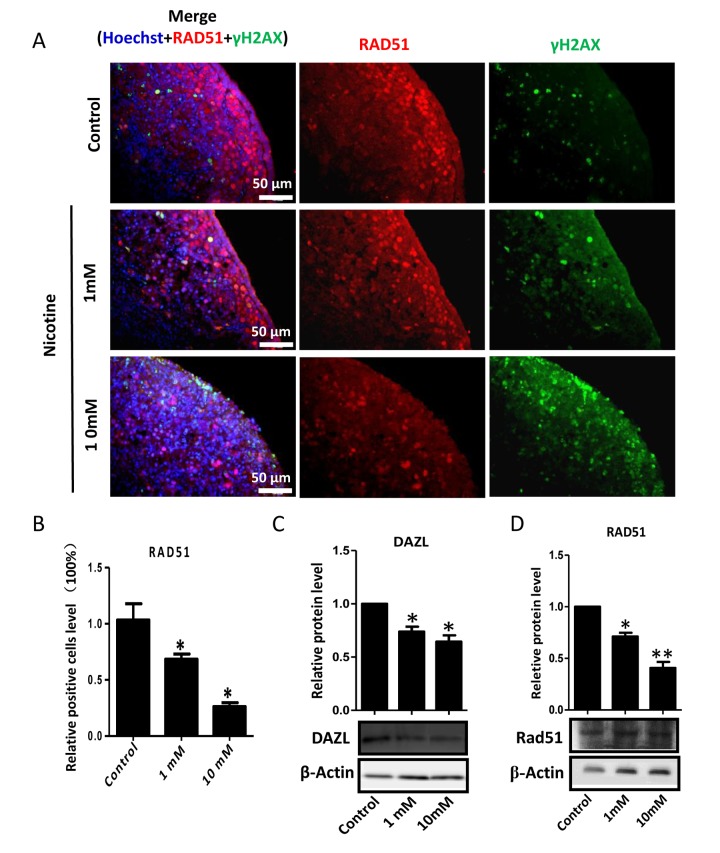
**Dose-dependent decrease or increase of the number of meiotic germ cells (RAD51) and of γH2AX positive cell, respectively, in nicotine treated fetal ovaries cultured for 4 days.** (**A**) Representative IF images of ovarian tissue sections for RAD51 and γH2AX; (**B**) Relative percentage of RAD51 positive cells of ovaries cultured without (control) and with 1mM or 10mM nicotine. (**C**) WB and relative densitometric analyses of DAZL amount in control and nicotine exposed ovaries. (**D**) Representative WB and relative densitometric analyses of RAD51 amount in control and nicotine exposed ovaries. All experiments were repeated at least three times. Results are presented as mean ± SD. (*) and (**) indicate significant (P < 0.05) and highly significant (P < 0.01) difference, respectively.

**Figure 7:**
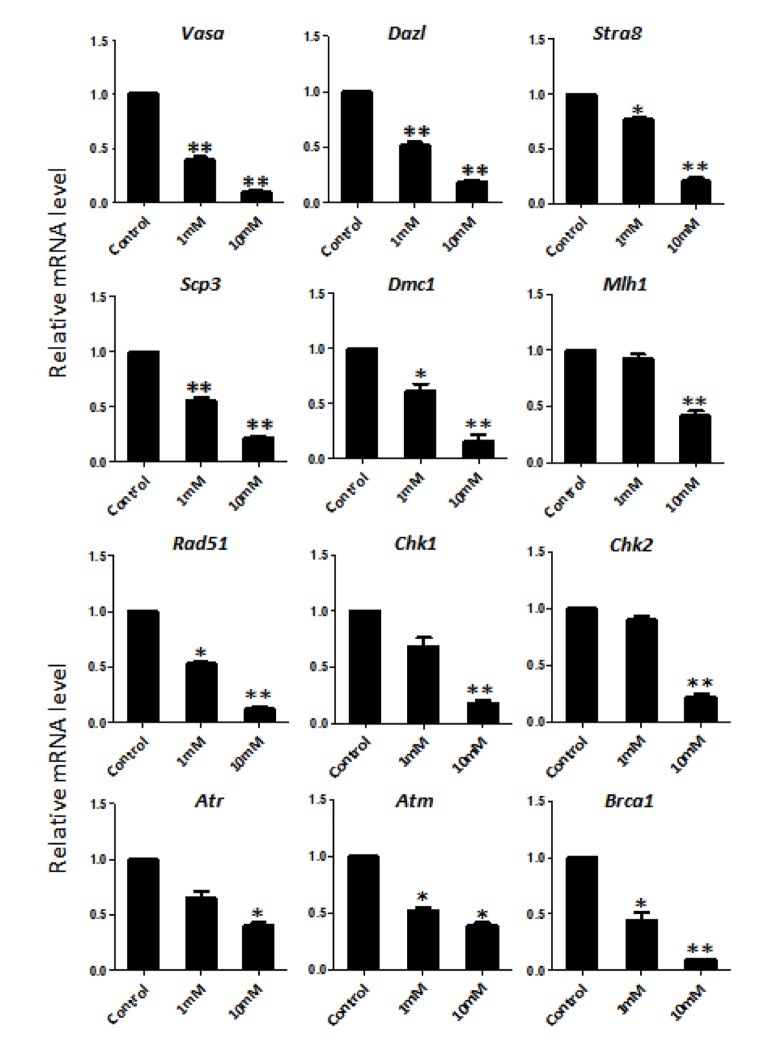
**Dose-dependent nicotine decrease of mRNAs of germ cell specific and meiotic genes in fetal ovaries cultured for 4 days evaluated by qRT-PCR.** The expression level was normalized to that of *Gapdh* gene. All experiments were repeated at least three times. Results are presented as mean ± SD. (*) and (**) indicate significant (P < 0.05) and highly significant (P < 0.01) difference, respectively.

In order to better characterize the nicotine cell target, we performed IF staining of tissue sections of 4 day cultured ovaries with antibodies against the germ cell specific RNA binding proteins VASA and DAZL, and the meiotic proteins SCP3, MLH1 and RAD51. The results showed a marked reduction of the number of positive cells up to 50% and 75% in comparison to controls for 1 mM and 10 mM nicotine, respectively (Figure 4A, C and Figure S3; Figure 4B, C; Figure 5A, C; Figure 5B, C; Figure 6A, B), suggesting oocytes were affected by the nicotine exposure. Further supporting the negative effect of nicotine on oocytes we observed decreased levels of DAZL and RAD51 proteins (Figure 6C, D) and of transcripts of *Stra8*, a gene encoding a protein crucial for entering into meiosis [47], and of *Atm*, *Atr*, *Chk1*, *Chk2, Brca1* genes, encoding proteins involved in meiosis associated processes such as homologous recombination and/or DNA repair, in the ovaries cultured in the presence of nicotine (Figure 7). IF with antibodies against ɤH2AX, a marker for DNA damage and repair [48-51], showed a nicotine dose and time dependent increase in the number of positive cells up to 3-5 fold in the ovaries incubated for 4 days in the presence of the higher nicotine concentrations (Figure 2B, C, and Figure S2). Nicotine appeared to cause DNA damage in both germ cells and somatic cells as IF double staining for VASA and ɤH2AX or for MLH1 or RAD51 and ɤH2AX, were not always overlapping (Figures 4A, 5B, 6A). IF double staining for MLH1 or RAD51 and ɤH2AX also showed that while the majority of the control MLH1 and RAD51 positive oocytes were negative for ɤH2AX, those remaining after exposure to nicotine were usually positive for the phosphorylated histone suggesting unrepaired DNA breaks. These results suggest that apoptosis in the ovarian cells was induced by nicotine due to increased DNA damage.

### Nicotine increased the oxidative stress in ovary cultured *in vitro*

**Figure 8:**
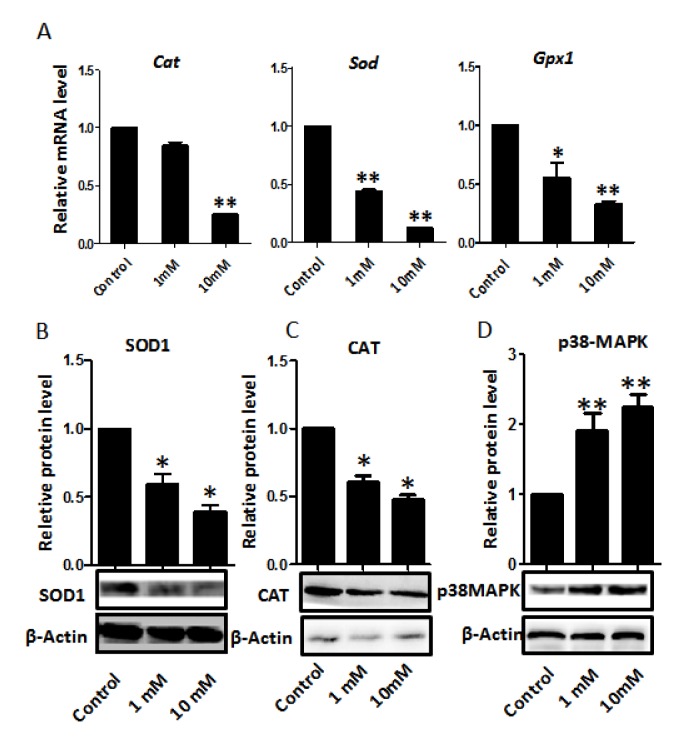
**Dose-dependent nicotine decrease of antioxidative enzyme gene transcripts in fetal ovaries cultured for 4 days.** (**A**) qRT-PCR for *Sod1*, *Cat* and *Gpx* mRNA levels in control and nicotine treated ovaries. The expression levels were normalized to that of *Gapdh* gene. (**B**) Representative WB and relative densitometric analyses of SOD1 proteins in control and nicotine treated ovaries. (**C**) WB and relative densitometric analyses of CAT amount in control and nicotine exposed ovaries. (**D**) The results of p38MAPK WB and relative densitometric analyses in control and nicotine exposed ovaries. All experiments were repeated at least three times. Results are presented as mean ± SD. (*) and (**) indicate significant (P < 0.05) and highly significant (P < 0.01) difference, respectively.

Since nicotine has been reported to induce oxidative stress in several tissues [1,34,52-54], and reactive oxygen species (ROS) associated with this process are frequent causes of DNA damage, we looked for markers of oxidative stress in the nicotine treated ovaries. We found significantly lower levels of mRNA for genes encoding antioxidant enzymes such as superoxide dismutase (*Sod*), catalase (*Cat*), and glutathione peroxidase (*Gpx*) in the nicotine treated ovaries cultured for 4 days that could cause or exacerbate ROS production (Figure 8). Moreover, in these ovaries, the levels of SOD1 and CAT proteins, evaluated by WB, were also decreased in comparison to controls (Figure 8). In this regard, it is to be mentioned that a decrease in the activities of SOD, CAT and GPX resulting in the increased generation of ROS has been reported in the blood of nicotine administrated rats [55]. In addition, the western blot showed that the p38-MAPK was increased in the nicotine treated group compared with the control group.

## DISCUSSION

In the present paper, we investigated the effect of nicotine on the human fetal ovary development *in vitro*, as there is limited information about this subject [56]. The *in vitro* ovary culture provides a useful and irreplaceable assay to obtain information about this crucial process during fetal oogenesis in human [8,57,58].

We found that the ovaries expressed the transcripts of various subunits of the nAChR and that exposure to nicotine for 4 day appeared to modulate the expression level of some of the transcripts in a significant manner (Figure 1). Receptors for nicotine including nAChRs are expressed by multiple cell types of diverse origins and functions including granulosa cells of adult rat ovaries and many of these cells synthesize and release acetylcholine [45,59]. It has also been well documented that up-or downregulation of nAChRs, following nicotine exposure, occurs in various cell types in a dose dependent manner (for a review, see Zhang et al., 2017) [59]. The unique functional and pharmacological properties of nAChRs are likely to contribute to highly specific local and tissue specific responses to circulating levels of nicotinic ligands. There are several ligands that through interacting with nAChRs potentially regulate the activity of specific nAChR subtypes. This allows regulation of a broad range of biological functions including cellular proliferation, apoptosis, migration, and signal transduction (for a review, see Zhang et al., 2017) [59]. The significance and physiological functions of nAChR expression in the fetal ovaries, reported to our knowledge by us for the first time in the present paper, are at the moment only speculative.

On the basis of the altered morphology observed in ovaries cultured in the presence of the higher concentration of nicotine (Figure 2), we hypothesised ongoing apoptosis in the ovarian cells. Nicotine has been shown to induce apoptosis in a variety of tissues [60] and exposure to the chemical during fetal, neonatal, and adult stages in female rats and mice induced altered morphology and increased apoptosis in the ovaries [44-46].

In this paper, we used TUNEL as a cell apoptosis marker. TUNEL highlights breaks in DNA that occur during the later stages of apoptosis. It is not a specific marker of apoptosis, but may offer insights into the state of DNA in labelled cells [61]. TUNEL was used successfully as a correlate of cell death in human oocytes by Modi et al. who found that 3-7% of oocytes were apoptotic between weeks 13-23 in normal ovaries, rising to >50% in Turner’s syndrome 45XO ovaries, where extensive prenatal loss of oocytes occurs [62]. Albamonte et al. also observed low levels (≦10%) of TUNEL-positive germ cells throughout the early second trimester, but they found a higher incidence (~20%) at 18-20 weeks [63]. Hartshorne et al. used TUNEL as a marker of cell apoptosis in oocytes at different stages of meiotic prophase I at known gestational ages [61]. In addition, Albamonte et al. demonstrates that apoptosis-inhibiting Bcl-2 protein and apoptosis-inducing BAX protein have different patterns of expression in the developing human ovary, they found Bcl-2 was detected from week 12 to 17 and became undetectable thereafter. Strong BAX signal was detected in oogonia and oocytes from week 12 until term [63].

For detecting apoptotic cells in follicles formed in fetal, neonatal, prepubertal, and adult ovaries Tsoulis et al. used activated caspase-3 as a biomarker. They found there was a significant increase in the proportion of activated caspase-3 immuno-positive cells, indicative of increased apoptosis in the fetal ovary as a result of a maternal high-fat diet [64]. In order to get more accurate results, we used TUNEL, Bcl-2/BAX and activated caspase-3 methods to investigate the extent of apoptosis in ovaries cultured with or without nicotine. Previously, nicotine has been shown to induce apoptosis in a variety of human tissues [60] and is associated with the activation of Caspase 3, TUNEL and increasing *Bax/Bcl-2* [65,66]. Consistent with our results, it has been shown that exposure to the nicotine during fetal, neonatal and adult age induces altered morphology and apoptosis in rodent ovaries. In particular, Petrik and coll [45] reported impaired folliculogenesis in the ovaries of offspring from mothers exposed to nicotine during pregnancy.

In conclusion, the results reported in the present paper support the notion that nicotine induced apoptosis, perhaps as a consequence of ROS induced DNA damage in the fetal ovarian cells, may be the cause of the reduction in the ovarian reserve and of the consequent precocious menopause in some patients associated with mothers smoking during pregnancy.

## MATERIALS AND METHODS

### Ethics statement

All procedures were approved by the Ethics Committee of Qingdao Agricultural University, and were in accordance with the agreement of the Ethics Committee at Qingdao Agricultural University (Agreement No. 2013-16).

### Collection of fetal ovaries

Human fetal ovaries at the second trimester (about 16 weeks of gestation) were obtained following medical termination of pregnancy. The informed written consent was obtained from all patients in accordance with national guidelines. Mifepristone (200 mg) and misoprostol treatment (800 µg) were used for termination of the pregnancy. None of the terminations were for reasons of fetal abnormality and the women were not smoking during the pregnancy period. Gestational age was determined by ultrasound examination before termination and confirmed by subsequent direct measurement (Table S1). The collected fetal ovaries were: (i) processed for *in vitro* culture, (ii) processed for histology or (iii) snap-frozen and stored at -70 °C for subsequent analysis.

### Culture of fetal ovaries and experimental design

In total, 12 female fetuses (24 ovaries) were included in this study. Each single human fetal ovary was cut longitudinally and transversely into 4 pieces and a pair of human fetal ovaries were divided into 8 groups. Group 1 to 4 were treated with nicotine (Sigma Chemical Co., 613207, St. Louis, MO, USA) at the concentration of 0 (control), 0.01, 0.1 and 0.5 mM, respectively; group 5 to 8 were treated with nicotine at the concentration of 0 (control), 1, 10 and 20 mM, respectively. The ovarian pieces were cultured on cell culture inserts (Invitrogen) in serum free medium [α-MEM supplemented with 1 × GlutaMAX and nonessential amino acids (Applied Biosystem), 2mM sodium pyruvate, 3mg/ml BSA Fraction V (Sigma-Aldrich, USA), and penicillin/ streptomycin/amphotericin B (Gibco, China)], as previously described [8,58]. Nicotine doses were selected according to previous *in vitro* experiments performed by Sudheer et al. [34] and Kim et al. [65]. The nicotine was freshly added to the culture medium at the indicated concentrations. Nicotine was dissolved in medium and pH was adjusted to 7.4. All ovaries were cultured for 2 or 4 days in a CO_2_ incubator at 37 °C. Medium change was performed every 24 hours and fresh nicotine added at every change. Each experiment was repeated three times.

### Immunofluorescence

Immunofluorescence (IF) was carried out on ovarian tissue sections as follows. Briefly, after fixation with 4% paraformaldehyde for 12 h, according to the standard histological procedures the ovarian pieces were processed for paraffin sectioning. Serial 5µm sections were heated at 60 °C for 2 h. After this the slides were rehydrated with a series of graded ethanol and washed with PBS 0.01 M sodium citrate were used for antigen retrieval at 95°C. After blocking for 1 hr with 10% BSA, the slides were incubated overnight at 4 °C with primary antibodies, anti: VASA (Abcam, ab13840, USA), SCP3 (NOVUS, NB300-232), DAZL (Abcam, ab34139), ɤH2AX (Sigma, SAB4501369), RAD51 (Abcam, ab202063), MLH1 (Abcam, ab92312), diluted to optimal concentrations. After thoroughly rinsing with TBS, secondary antibodies (CY3, A0516, Beyotime; FITC, A0568, Beyotime, China) diluted 1:200 were applied at 37 °C for 1h in the dark. After washing three times with PBS, the samples were incubated with Hoechst33342 (Solarbio, China) and observed under a fluorescence microscope. Positive cells were scored as previously described [67]. Negative controls were performed omitting the primary antibodies after the blocking procedure (not shown).

### Quantitative real-time PCR

For quantitative real-time PCR ovarian pieces were collected. An RNA extraction kit was used for total RNA extraction according to the manufacturer’s protocol (TaKaRa, Dalian, China). After reverse transcription into cDNA (TransScript One-Step, Beijing, China), a quantitative real-time PCR experiment was done with SYBR Green I Master (Roche, 04887352001, Germany). Table S2 showed the primers (designed and purchased from Invitrogen, Shanghai). The gene expression were normalized to *Gadph* and analyzed using the formula: 2^^-(target gene CT value –reference gene CT value)^.

### Western blotting

For western blotting (WB) analysis of total protein extracts were obtained from tissues using RIPA lysis solution (Beyotime, P00113B). After separation by SDS-PAGE, the proteins were electrophoretically transferred onto a polyvinylidene fluoride membrane at 200mA for 5 h using Trans-Blot apparatus. The membranes were blocked with 5% BSA at 4 °C for 12 h, after washing with TBST three times they were incubated with the appropriate primary antibodies for1 h at 37 °C, anti: DAZL (Abcam, ab34139), RAD51 (Abcam, ab63801), BAX (Beyotime, AB026), Bcl-2 (Beyotime, ab112-1), SOD1 (Abcam, ab20926), CAT (Abcam, ab16731), pMAPK (Abcam ab197348). After washing as described above the membrane was incubated with the secondary antibodies (horseradish peroxidase (HRP)-conjugated goat anti-rabbit IgG (Beyotime, A0208) or goat anti-mouse IgG (Beyotime, A0126) for 1 h at 37 °C. Subsequently, the membrane was washed three times with TBST, and a BeyoECL plus Kit (Beyotime, P0018) was used for exposure. Densitometric analyses were performed using IPWIN software.

### TUNEL and Activated Caspase 3 staining

Apoptosis was evaluated in paraffin tissue sections of ovaries using the Bright Red Apoptosis Detect Kit for terminal deoxynucleotide transferase dUTP nick end labelling (TUNEL) (Vazyme, A113-02, Nanjing, China). Briefly, the ovary sections were heated at 60 °C for 2 h and after washing with xylene passed through a graded series of ethanol for rehydration and washed in PBS. Sections were incubated in Proteinase K for 15 min, then washed twice with PBS. The samples were incubated in the dark for 60 min at 37 °C in 100 μl TUNEL reaction mixture and counterstained with Hoechst33342.

Activated Caspase 3 was identified used Immunofluorescent staining and the ovaries were processed as described above. Briefly, after blocking for 1 hr with 10% BSA, the slides were incubated overnight at 4 °C with primary antibodies, anti: Caspase3 (Abcam, ab2302), diluted to optimal concentration. After thoroughly rinsing with TBS secondary antibodies (CY3, A0516, Beyotime) diluted 1:200 were applied at 37 °C for 1h in the dark. After washing three times with PBS, the samples were incubated with Hoechst33342 (Solarbio, China) and observed under a fluorescent microscope.

### Statistical analysis

Results are represented as mean ± SD. Statistical significance among means was determined with GraphPad Prism analysis software (Graph-Pad Software, San Diego, CA, USA) by Student’s t-test or ANOVA followed by Tukey test for multiple comparisons. Comparisons were considered significant at P < 0.05 and highly significant at P < 0.01.

## Supplementary Material

Supplementary File
