# Correction for: Nicotine exposure impairs germ cell development in human fetal ovaries cultured *in vitro*

**DOI:** 10.18632/aging.204392

**Published:** 2022-11-15

**Authors:** Shun-Feng Cheng, Xun-Si Qin, Ze-Li Han, Xiao-Feng Sun, Yan-Ni Feng, Fan Yang, Wei Ge, Lan Li, Yong Zhao, Massimo De Felici, Shu-Hua Zou, Yi Zhou, Wei Shen

**Affiliations:** 1 College of Life Sciences, Institute of Reproductive Sciences, Qingdao Agricultural University, Qingdao, 266109, China; 2 The First Affiliated Hospital of Chinese PLA General Hospital, Beijing, 100039, China; 3 Department of Biomedicine and Prevention, University of Rome ‘Tor Vergata’, Rome, 00133, Italy; 4 Center for Reproductive Medicine, Qingdao Women’s and Children’s Hospital, Qingdao University, Qingdao, 266034, China

**This article has been corrected:** The authors recently found the errors in the **Figures 2A** and **4B**. In **Figure 2A**, the images of cultured ovaries in the “Control” and “1 mM Nicotine” groups are the same because the authors accidentally placed a “Control” group image instead of “1 mM Nicotine” group image. They replaced the incorrect image with an original image of the “1 mM Nicotine” group from the original set of experiments. In **Figure 4B**, the fluorescent image of the “10 mM Nicotine” group stained with DAZL was incorrect and did not correspond to the “Merged” image. The authors replaced this image with a corresponding image from the original data. These alterations do not affect the results or conclusions of this work. The authors would like to apologize for any inconvenience caused.

New **Figures 2** and **4** are presented below.

**Figure 2:**
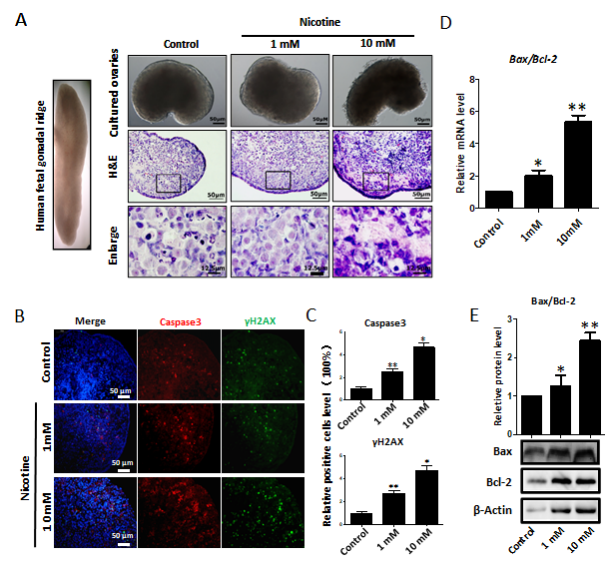
**Dose-dependent nicotine induction of apoptosis in fetal ovaries cultured for 4 days.** (**A**) Ovaries cultured without (control) and with 1 mM or 10 mM nicotine; note altered ovary morphology at 10mM nicotine and representative H&E histological sections of the ovaries; (**B**) IF for Caspase3 and ɤH2AX in tissue sections of ovaries cultured without (control) and with 1mM or 10mM nicotine; (**C**) Relative percentage of Caspase3 and ɤH2AX positive cells in ovaries cultured without (control) and with 1mM or 10mM nicotine; (**D**) *Bax/Bcl2* mRNA ratio in samples extracts from ovaries cultured without (control) and with 1mM or 10mM nicotine. The expression level was normalized to that of *Gapdh*. (**E**) Increased BAX/BCL2 protein ratio in nicotine exposed ovaries in comparison with control. All experiments were repeated at least three times. Changes are presented as mean ± SD. (*) and (**) indicate significant (P < 0.05) and highly significant (P < 0.01) difference, respectively.

**Figure 4:**
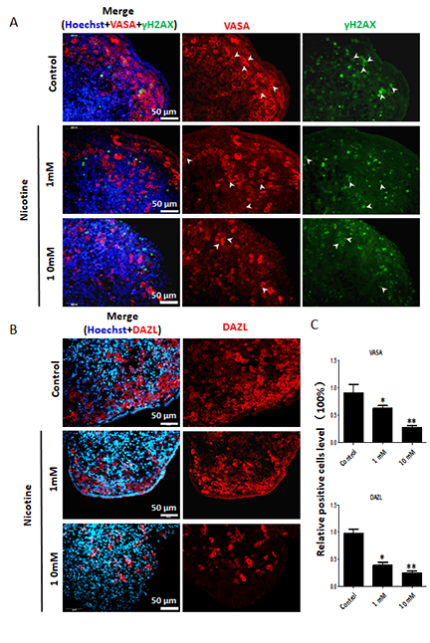
**Dose-dependent decrease or increase of the number of germ cell (VASA, DAZL) and of γH2AX positive cells, respectively, in nicotine treated fetal ovaries cultured for 4 days.** (**A**) Representative IF images of ovarian tissue sections for VASA and γH2AX; note that only a subset of the γH2AX positive cells were also VASA positive (arrow heads); (**B**) Representative IF images of ovarian tissue sections for DAZL; (**C**) Relative percentage of VASA and DAZL positive cells of ovaries cultured without (control) and with 1mM or 10mM nicotine. All experiments were repeated at least three times. (*) and (**) indicate significant (P < 0.05) and highly significant (P < 0.01) difference, respectively.

