# Supplementary material for: Nicotine exposure impairs germ cell development in human fetal ovaries cultured in vitro
Source: Aging (Albany NY). 2018 Jul 12;10(7):1556–74. doi: 10.18632/aging.101492 (PMC6075447; doi:10.18632/aging.101492)
Supplement: Supplementary File [file aging-10-101492-s001.pdf]

SUPPLEMENTARY MATERIAL

Supplementary Table S1. Fetal characteristics examined in this study.

Fetal characteristics (n=12)	Measurements
Age (weeks)	16.4±0.6
Weight (g)	126±8
Biparietal diameter（mm）	35.1±4.9
Femur length (mm)	22±4.2
Abdominal circumference (mm)	105.2±17.2
Fetal head circumference (mm)	123±9

Supplementary Table S2. Primers Used for Quantitative Real-Time PCR.

Genes	Sequences of primers	Production (bp)	Genbank
Sod1	F:5′-GGGGAAGCATTAAAGGACTGA-3′<br>R:5′-CCACCGTGTTTTCTGGATAGA-3′	124	NM_001005735.1
Cat	F:5′-GCTCCAAATTACTACCCCAACA-3′<br>R: 5′-ATAGAATGCCCGCACCTGA-3′	141	NM_001752.3
Gpx1	F: 5′-AGTCGGTGTATGCCTTCTCG-3′<br>R:5′-AGCTCGTTCATCTGGGTGTAGT-3′	145	NM_000581.2
Atr	F: 5′-GCTCTTTTGGATGTGCTTGG-3′<br>R:5′-TCCTCAGTCTGTTTTGGTGCT-3′	218	XM_011512927.1
Brca1	F: 5′-CACAGTCGGGAAACAAGCA-3′<br>R: 5′-CTGACCAACCACAGGAAAGC-3′	288	NM_007294.3
Rad51	F: 5′-CCCATTTCACGGTTAGAGCA-3′<br>R:5′-TCTCAATTCCACCTGTAGTCCC-3′	289	NM_001164269.1
Atm	F: 5′-GACAATCATCACCAAGTTCGC-3′<br>R:5′-TCGCAGATAGGGCTACAGGA-3′	269	XM_011542846.1
Bax	F: 5′-GTCGCCCTTTTCTACTTTGC-3′<br>R: 5′-GGGACATCAGTCGCTTCAGT-3′	184	XM_006723314.2
Bcl2	F: 5′-TTGAGTTCGGTGGGGTCAT-3′<br>R:5′-CAGCCAGGAGAAATCAAACAG-3′	194	NM_000633.2
Mlh1	F: 5′-GAGGAAGGGAACCTGATTGG-3′<br>R: 5′-CGGATGGAATAGAACATAGCG-3′	167	XM_011533727.1
Vasa	F: 5′-AGCTGGGACATTCAATTCGAC-3′<br>R:5′-GTTTGGCTGCGTTCCTTTGAT-3′	220	NM_001166534.1
Scp3	F: 5′-AAATCTGGGAAGCCGTCTGT-3′<br>R:5′-AACTCCAACTCCTTCCAGCA-3′	207	NM_001177949.1
Chk1	F 5′-CCTTTGTGGAAGACTGGGACT-3′<br>R:5′-ACAATCTTCACTGCGACTGCT-3′	109	NM_001244846.1
Chk2	F:5′-AAAGTGCTGGGATAAGAGGTGT-3′<br>R:5′-TCCCTGAAAATCCGAAAGTG-3′	181	NM_001005735.1
Dazl	F:5′-GACTAATCCAAACACTGAAACTTAT-3′<br>R:5′-TACAGTGGTAGTTAACAGCTGAATA-3′	221	NM_001190811.1
nAChR-α1	F:5′-GCTCTGTCGTGGCCATCAA-3′<br>R:5′-CCGGAAAGCGACCAGCCAGA-3′	40	NM_000079.3
nAChR-α2	F:5′-GTGGAGGAGGAGGACAGA-3′<br>R:5′-CTTCTGCATGTGGGGTGATA-3′	156	NM_000742.3

nAChR-α3	F:5′-CAGAGTCCAAAGGCTGCAAG-3′<br>R:5′-AGAGAGGGACAGCACAGCAT-3′	149	NM_000743.4
nAChR-α4	F:5′-CTCACCGTCCTTCTGTGTC-3′<br>R:5′-CTGGCTTTCTCAGCTTCCAG-3′	110	NM_000744.5
nAChR-α5	F:5′-CTTCACACGCTTCCCAAACT-3′<br>R:5′-CTTCAACAACCTCACGGACA-3′	187	NM_000745.3
nAChR-α6	F:5′-TCCATCGTGGTGACTGTGT-3′<br>R:5′-AGGCCACCTCATCAGCAG-3′	126	NM_001199279.1
nAChR-α7	F:5′-GTACGCTGGTTTCCCTTTGA-3′<br>R:5′-CCACTAGGTCCCATTCTC-3′	139	NM_000746.4
nAChR-α9	F:5′-GAAAGCAGCCAGGAACAAAG-3′<br>R:5′-GCACTTGGCGATGTACTCAA-3′	157	NM_017581.2
nAChR-α10	F:5′-ACACAAGTGCCCTGAGACCT-3′<br>R:5′-TCCCATCGTAGGTAGGCATC-3′	160	NM_020402.2
nAChR-β1	F:5′-CTACGACAGCTCGGAGGTCA-3′<br>R:5′-GCAGGTTGAGAACCACGACA-3′	479	NM_000747.2
nAChR-β2	F:5′-GGCATGTACGAGGTGTCCTT-3′<br>R:5′-CACCTCACTCTTCAGCACCA-3′	200	NM_000748.2
nAChR-β3	F:5′-AACAGTTCCGTTTGATTTCACGAT-3′<br>R:5′-CCCTGATGACCAAGGTCATC-3′	41	NM_000749.3
nAChR-β4	F:5′-TCCCTGGTCCTTTTCTTCCT-3′<br>R:5′-TGCAGCTTGATGGAGATGAG-3′	160	NM_000750.3
nAChR-γ	F:5′-CGCCTGCTCTATCTCAGTCA-3′<br>R:5′-GGAGACATTGAGCACAACCA-3′	547	NM_002046.3
Gapdh	F:5′-GAGTCAACGGATTTGGTCGT-3′<br>R:5′-TTGATTTTGGAGGGATCTCG-3′	238	NM_002046.4

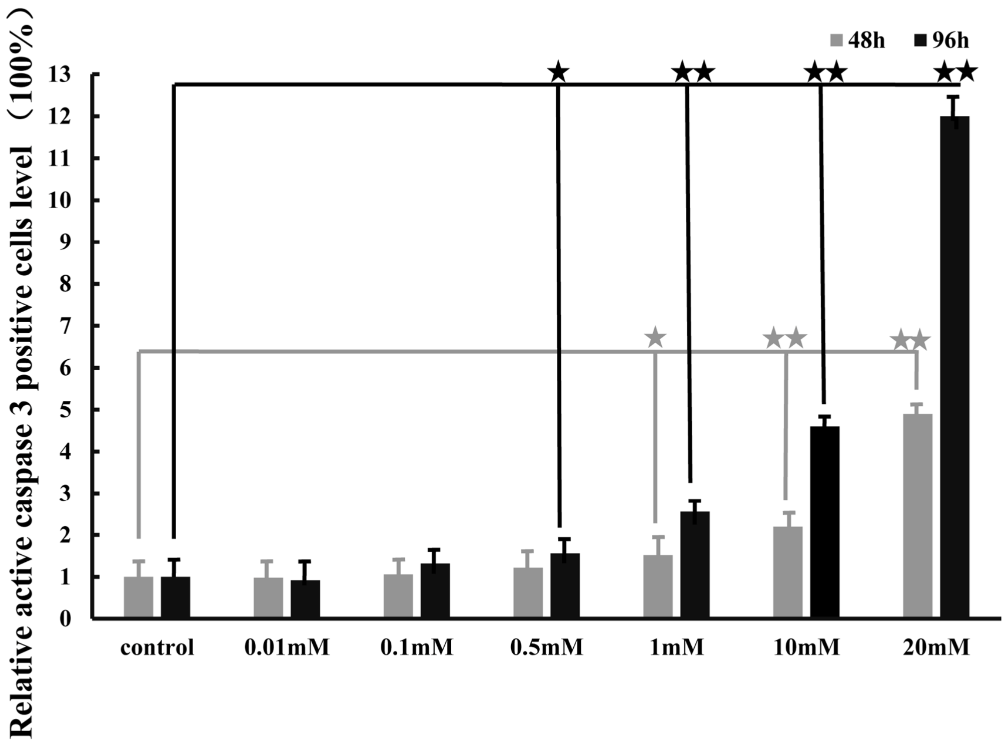

Supplementary Figure S1. Relativer ration of Caspase3 positive cells in ovaries after 48 h or 96 h incubation in the presence of different concentrations of nicotine. Results are presented as the mean± SD. All experiments were repeated at least three times. (*) and (**) indicate significant (P<0.05) and highly significant (P < 0.01) difference, respectively.

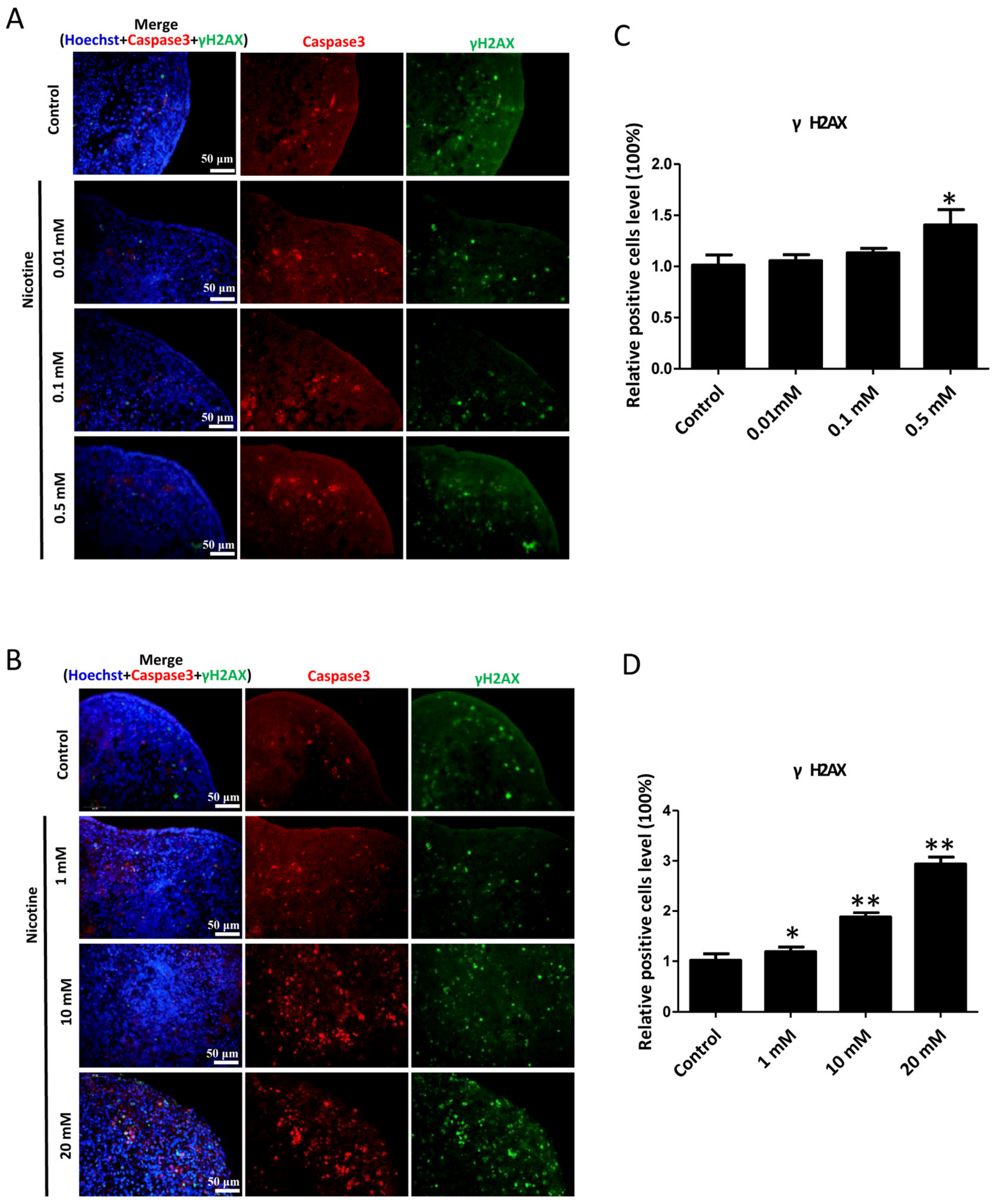

Supplementary Figure S2. Representative IF images of tissue sections for active Caspase 3 and ɣH2AX of ovaries cultured for 4 days (**A**) and 2 days (**B**) in the presence of increasing nicotine concentrations. (**C**) Relativer ration of ɣH2AX positive cells after 4 days of culture; (**D**). Relativer ration of ɣH2AX positive cells after 2 days of culture. All experiments were repeated at least three times. (*) and (**) indicate significant ($P < 0.05$) and highly significant ($P<0.01$) difference, respectively.

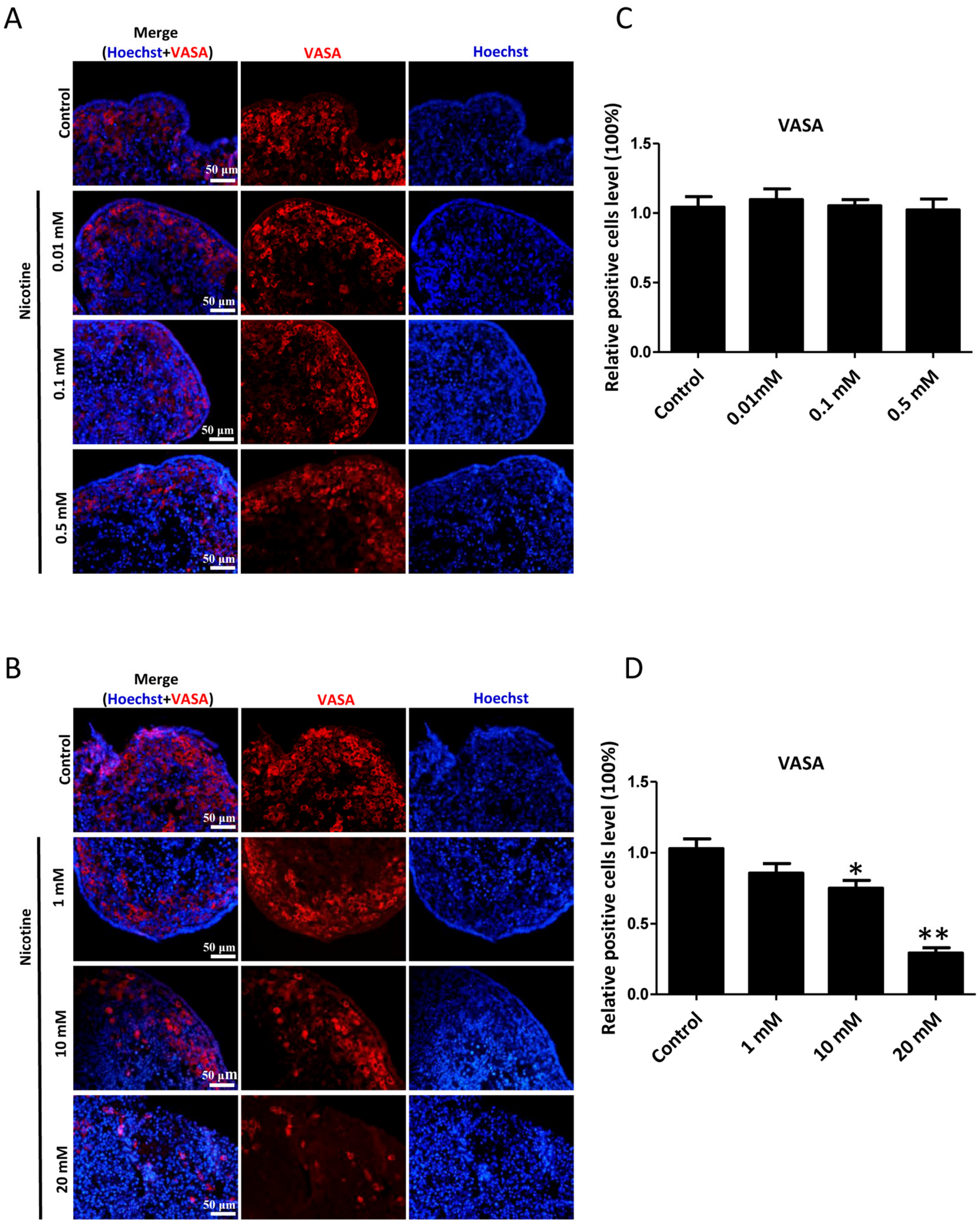

Supplementary Figure S3. Representative IF images of tissue sections for VASA positive cells (germ cells) in human fetal ovaries cultured for 4 days (**A**) and 2 days (**B**) in the presence of increasing nicotine concentrations. (**C**) Relativer ration of VASA positive cells after 4 days of culture; (**D**) Relativer ration of VASA positive cells after 2 days of culture. All experiments were repeated at least three times. (*) and (**) indicate significant (P<0.05) and highly significant (P<0.01) difference, respectively.
